# Recyclable and Degradable Poly(vinyl alcohol)/Betaine-Based Deep Eutectic Polymer Dry Gel Plastics with a High Mechanical Strength

**DOI:** 10.3390/gels11060421

**Published:** 2025-05-31

**Authors:** Hanyu Zhao, Ying Jia, Ling Cai, Xiaochun Wang, Minghui He, Guangxue Chen

**Affiliations:** 1 State Key Laboratory of Pulp and Paper Engineering, School of Light Industry and Engineering, South China University of Technology, Guangzhou 510640, China; 2 Division of Engineering in Medicine, Department of Medicine, Brigham and Women’s Hospital, Harvard Medical School, Cambridge, MA 02139, USA; 3 Guangdong Province Filtration and Wet Nonwoven Composite Materials Engineering Technology Research Center, Guangzhou 510640, China

**Keywords:** deep eutectic polymer, dry gel, polymer, biodegradable

## Abstract

Most existing polymer plastics are nonreusable and also exhibit poor biocompatibility and a poor mechanical strength–tensile strain balance. Herein, using deep eutectic polymers, we prepare reusable hydrophilic supramolecular dry gel plastics with balanced stress–strain characteristics through the hydrogen bonding of poly(vinyl alcohol) (PVA) with betaine (Bta). As PVA exhibits crystalline stiffness and abundant hydrogen-bonding sites, it is employed as a network backbone in the proposed deep eutectic supramolecular polymers. In the prepared PVA/Bta dry gel plastics, PVA and Bta are dynamically and physically crosslinked through high-density hydrogen bonding, resulting in a yield strength of ~109 MPa and toughness of up to ~210.92 MJ m^−3^. In addition, these plastics can be recycled at least five times in an aqueous environment while maintaining a mechanical strength of 100 MPa. Furthermore, the proposed polymers exhibit high transparency (92%) in the visible spectrum. We expect these polymers to be used in synthesizing biodegradable dry gel plastics, as well as to lead to the development of recyclable deep eutectic PVA/Bta polymers with remarkable strength.

## 1. Introduction

Plastics are lightweight and strong materials that have become a staple in daily life owing to their mechanical properties [1,2,3,4,5]. Currently, most commercial plastics are petroleum-based thermoset polymers. The strong intermolecular carbon–carbon and carbon–hydrogen bonding structures of these plastics are difficult to break at ordinary temperatures, rendering their decomposition in natural environments difficult [6,7,8,9]. Thus, biodegradable plastics have been developed as a new-generation alternative to nonbiodegradable plastics to address the problem of plastic pollution [10,11]. In particular, polycaprolactone and polylactic acid can be degraded into smaller molecules by microorganisms in an appropriate environment; however, achieving the complete degradation of these plastics under natural conditions remains challenging because they break into tiny fragments, which poses another potential threat to the ecological security of soil ecosystems [12,13,14]. Moreover, the high cost of biodegradable plastics limits their widespread use. Therefore, green alternatives that are water soluble and easy to recycle are gradually attracting widespread attention [15,16,17]. For instance, Zhang et al. [18] employed chitosan to produce entirely biogenic water-soluble polymers with outstanding mechanical properties (tensile strength: 62.9 MPa) and high antibacterial activity. Tian et al. [19] reported a composite of poly(vinyl alcohol) (PVA) and waste cottonseed hulls with reasonably good mechanical (maximum tensile strength: 10.3 MPa) and thermal (degradation temperature: ~250 °C) properties; notably, the solubility of PVA in water ensured the environmental friendliness of the composite, with the PVA matrix dissolving in soil within 10 d without any adverse effects on plants. However, despite numerous reports on hydrophilic plastics, the mechanical strength and tensile properties of these reported materials after recycling do not satisfy the durability requirements of real-life production. To overcome these shortcomings, a plastic that can be recycled without the excessive loss of mechanical properties must be developed.

Studies have reported several sustainable, biodegradable, low-toxicity, and solution-stabilized deep eutectic polymers in air [20,21,22]. Different hydrogen-bonded crosslinking networks can be constructed through monomer design to endow deep eutectic polymers with properties suitable for different use scenarios. Li et al. [23] prepared a polymerizable deep eutectic solvent (PDES) system comprising acrylamide and maleic acid; the abundant hydrogen bonds between the PDES chains in the involved elastomer could be easily broken and reorganized, endowing the system with excellent self-repair capabilities at low temperatures (−23 °C) without requiring any external stimuli. These properties make such elastomers promising for application in flexible electronic devices operating at low temperatures. Based on a dual-network design, Zhao et al. [24] combined a stretchable poly(acrylic acid (AA)–choline chloride (ChCl))-type supramolecular deep eutectic polymer network with a brittle poly(vinylpyrrolidone) network to prepare high-strength, stretchable, ion-conducting, and biocompatible liquid-free ionic conductors. However, such ionic conductors are usually prepared from monomers through crosslinking, polymerization, and ultraviolet (UV) curing, inevitably leading to residues such as acrylic acid, which are very harmful to aqueous organisms [25,26,27,28].

To address the aforementioned issue, herein, we prepared functional, water-compatible, and deep eutectic composites from polymers [29,30,31]. Notably, PVA is a water-soluble, nontoxic, inexpensive, and biodegradable synthetic polymer possessing abundant hydroxyl groups and thus may function as a hydrogen bond donor (HBD) [1,30,32,33,34,35,36,37,38,39,40,41]. Additionally, PVA is completely degraded to CO_2_ and H_2_O by microbes in soil and wastewater [11,42,43,44,45,46,47,48,49]. Meanwhile, betaine (Bta) is a natural compound that functions as a hydrogen bond acceptor (HBA) and may be derived from foods such as wheat and spinach [50,51,52]. Through hydrogen bonding between PVA and Bta, we formed a deep eutectic supramolecular system exhibiting considerably better mechanical properties than single-component PVA films, showing four times greater elongation and higher tensile strength. Further, the prepared PVA/Bta dry gel plastics (PALBs) were biodegradable in soil, exhibited a UV transmittance rate of >90% within a wavelength band of 400–800 nm, and could be recycled and reused.

## 2. Results and Discussion

**Figure 1:**
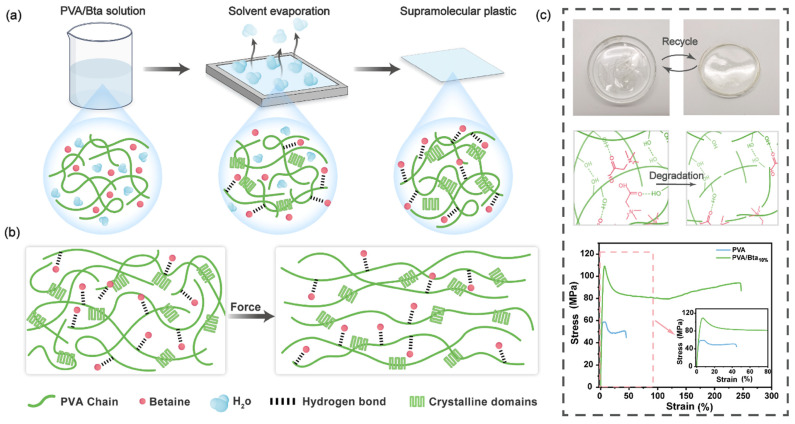
(**a**) Schematic of the dry gel plastic preparation process; (**b**) the force triggering mechanism; and (**c**) the supramolecular interactions of tough, recyclable supramolecular dry gel plastics.

PVA was chosen as the main HBD for preparing the supramolecular dry gel plastics because of its abundant hydroxyl groups and intermolecular hydrogen bonds. In the supramolecular system, two types of hydrogen bonds exist: those between the hydroxyl groups (OH) in the molecular chain of PVA and those between the –OH groups in PVA and the carbonyl group (C=O) in Bta. The electron cloud distribution of the C=O bond is shifted toward the oxygen atom. Thus, oxygen atoms in the carbonyl group of Bta form hydrogen bonds with –OH in PVA [53]. The prepared plastics are denoted as PVA/Bta*x*, where *x* represents the mass fraction of Bta in the binary blend. Table S1 presents the composition of the PVA/Bta blend.These dynamic systems are formed through hydrogen bonding between PVA and Bta (Figure 1 and Figure S1). Owing to the abundance of hydrogen-bonding sites and crystalline stiffness, PVA acts as a reinforcing phase, as well as achieving strong interfacial cohesion. The dynamic network formed by the HBA and HBD imparts desirable toughness to the supramolecular plastics through the reconstruction of dynamic hydrogen bonds.

### 2.1. Mechanical Performance of the PVA/Bta Dry Gel Plastics

**Figure 2:**
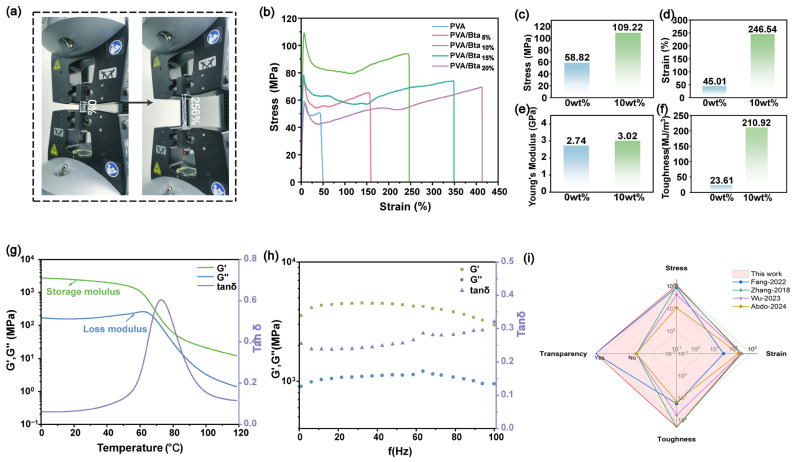
(**a**) Physical schematic of supramolecular dry gel plastics in the tensile state, (**b**) stress–strain curves for supramolecular dry gel plastics with 0–20% Bta mass ratios, (**c**–**f**) tensile stress–strain curves for PVA and PVA/Bta10% and corresponding property comparisons, (**g**) plot of PVA/Bta storage modulus versus temperature recorded via DMA at a frequency of 1 Hz, (**h**) plot of PVA/Bta energy storage modulus versus loss modulus versus frequency (0–100 Hz) recorded via DMA at a strain of 1%, and (**i**) comparison of mechanical properties between the prepared PVA/Bta deep eutectic polymer dry gel plastics and recently reported plastics [1,47,54,55].

Because of the strong hydrogen bonds between the system network, all the prepared plastics exhibit outstanding mechanical strength and tensile characteristics. The plastics are made up of two components: PVA and Bta. The crystalline zone of PVA exhibits well-organized molecular chains, a greater density than the amorphous zone, and a strain that increases with an increasing Bta content. This is because the application of an external force to the plastics results in the destruction of the crystalline structure of the system, allowing the molecular chains to move more freely and thereby releasing a force. Notably, the plastic with the 10% Bta mass ratio shows a tensile strength of up to 109 MPa and a strain at break of 246% (Figure 2a–d). Additionally, films with 10 wt% Bta exhibit the highest toughness (up to 210.92 MJ m^−3^) and Young’s modulus (up to 3.02 GPa), as shown in Figure 2e,f. Although the stresses are not as low as those of pure PVA plastics, they start decreasing as the amount of Bta is increased over 10% (Figure S2 and Figure 2b). Thus, Bta introduction improves the mechanical properties of PVA, attributed to the physical crosslinking and molecular chain entanglement between the PVA chains and Bta. The physical crosslinking and molecular chain entanglement are reversible. Notably, as shown in Figure 2i, the comprehensive mechanical performance of the prepared PVA/Bta deep eutectic polymer plastics is also considerably better than those of most previously reported reusable hydrophilic plastics.

Over the broad temperature range of 0–60 °C, the storage modulus and loss modulus of the prepared supramolecular polymer plastics exhibit similar variation trends (Figure 2g). According to the DMA results, the energy storage modulus of the supramolecular polymers decreases with increasing temperature. At 100 °C, they sustain a strength of 21 MPa, which is greater than the loss modulus over the studied temperature range. The energy storage modulus rapidly decreases in the vicinity of *T*_g_ (temperature corresponding to the extreme value of the loss factor). This is because the polymer transitions from the glassy to the highly elastic state as the temperature increases, with the dissociation of hydrogen bonds allowing the single-chain segments to move more freely and causing a rapid decrease in the storage modulus. Simultaneously, the relative slip between the polymer chains decreases, resulting in a decrease in the amount of energy lost as thermal energy and a decrease in the loss factor, which increases the elasticity of the supramolecular plastics. The *T*_g_ values obtained via DMA for various supramolecular dry gel plastics follow the same trend as those obtained through DSC. Figure 2h presents the plots of *G*′ and *G*″ versus frequency over a frequency range of 0–100 Hz, where *G*′ represents the stiff behavior and *G*″ represents the viscous behavior. In these graphs, *G*′ is always higher than *G*″, indicating the stiff behavior of the polymer network, consistent with the high Young’s moduli obtained through mechanical tensile tests.

### 2.2. Synthesis and Characterization of the PVA/Bta Dry Gel Plastics

**Figure 3:**
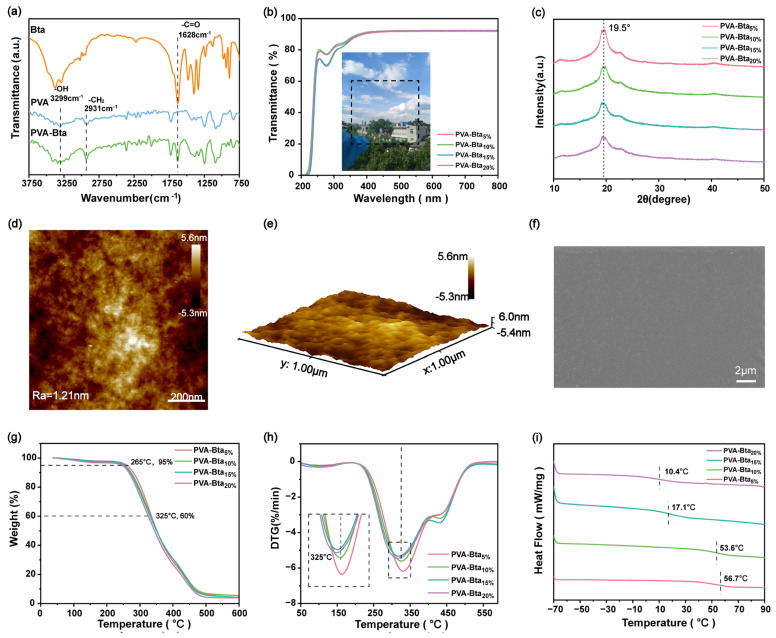
(**a**) FTIR spectra of each component of PVA/Bta dry gel plastics; (**b**) UV transmission curves; (**c**) X-ray diffraction (XRD) curves; (**d**,**e**) atomic force microscopy images of the PVA/Bta dry gel plastics; (**f**) scanning electron microscopy images of the PVA/Bta films; (**g**,**h**) thermogravimetry and differential thermogravimetry curves, respectively; and (**i**) differential scanning calorimetry (DSC) curve of the PVA/Bta supramolecular dry gel plastics.

The results obtained using DSC can provide direct evidence of the preparation of DESs. The melting point of a deep eutectic solvent should be lower than that of its components [56,57]. Figure S3a shows the DSC curves of the PVA/Bta supramolecular plastics. The melting point (*T*_m_) of the resulting sample is only around 163 °C, which is lower than those of PVA (*T*_m_ = 194 °C) and Bta (*T*_m_ = 301 °C). As shown in Figure S3b and Figure 3b the FTIR peaks at 1628 and 3299 cm^−1^ indicate that the carbonyl (C=O) bond in Bta and the hydroxyl group (OH) in PVA were not destroyed. Furthermore, the intensity of the peak at 1628 cm^−1^ and the width of the peak at 3299 cm^−1^ increase with increasing Bta content (Figure 3a and Figure S3a). Additionally, no new distinctive peaks appear in the plastics, confirming that the systems were formed solely via physical crosslinking through noncovalent bonds. Notably, some peaks are blue-shifted in the systems, confirming the formation of hydrogen bonds. The ^1^H NMR pattern of PVA/Bta and the individual peaks of PVA and Bta are shown in Figure S3c.

To confirm the presence of hydrogen bonds between PVA and Bta more intuitively, we simulated the interactions between them using the ball-and-stick model (the model was constructed using quantum chemistry methods). For convenience, the PVA hexamer was used; the obtained results are shown in Figure S4 and Table S2. The simulation results show that hydrogen bonding and electrostatic interactions are the primary polymerization drivers [30,31]. Macroscopically, the prepared dry gel plastics are highly transparent in a wavelength range of 400–800 nm (Figure 3b). In pure PVA plastics, the hydrogen bonds between the hydroxyl groups of the PVA main chain result in a diffraction peak at 2θ = 19.5°, indicating a semicrystalline structure (Figure 3c). Therefore, in the PVA/Bta system, the same diffraction peaks are observed as in PVA, but they gradually become broader with the addition of Bta, which reflects the formation of hydrogen-bonding forces in the system. The surface of the plastic films is flat on the micrometer scale, and the roughness is ~11.4 nm, which is in line with the requirements of practical applications (Figure 3d–f).

Figure 3g,h and Table S3 show some key parameters in the thermogravimetric analysis. It can be seen that the addition of Bta has an impact on plastics. The thermal stability has been somewhat weakened. After adding Bta, the T_onset_ of the PALBs shifted towards lower temperatures. Due to the increase in Bta content, it interacts with PVA chains to form new crosslinks, weakening the intramolecular hydrogen bonds of the original PVA chains and disrupting the ordered crystalline parts. However, this increases the flexibility of the molecular chains. From Table S3, it can be concluded that the prepared PALBs can be safely used at 210 °C. The glass transition temperature (*T*_g_) of the supramolecular dry gel plastics with different Bta contents was determined via DSC to investigate the effect of hydrogen-bonding interaction energy on the flexibility of polymer chains in PVA/Bta. Interactions between polymer chains usually reduce chain activity. The *T*_g_ of PVA is ~90 °C because of the strong hydrogen bonds between its polymer chains. At the same time, the *T*_g_ of PVA/Bta supramolecular plastics is lower because as an HBA, Bta weakens the interactions between PVA molecular chains owing to the formation of hydrogen bonds between Bta and PVA. This results in an increase in the flexibility of the PVA polymer chains in PVA/Bta supramolecular plastics, manifested in the Tg of PVA/Bta supramolecular plastics (approximately 10–70 °C) being lower than the Tg of PVA (approximately 90 °C).

### 2.3. Recyclability of PVA/Bta Dry Gel Plastics

**Figure 4:**
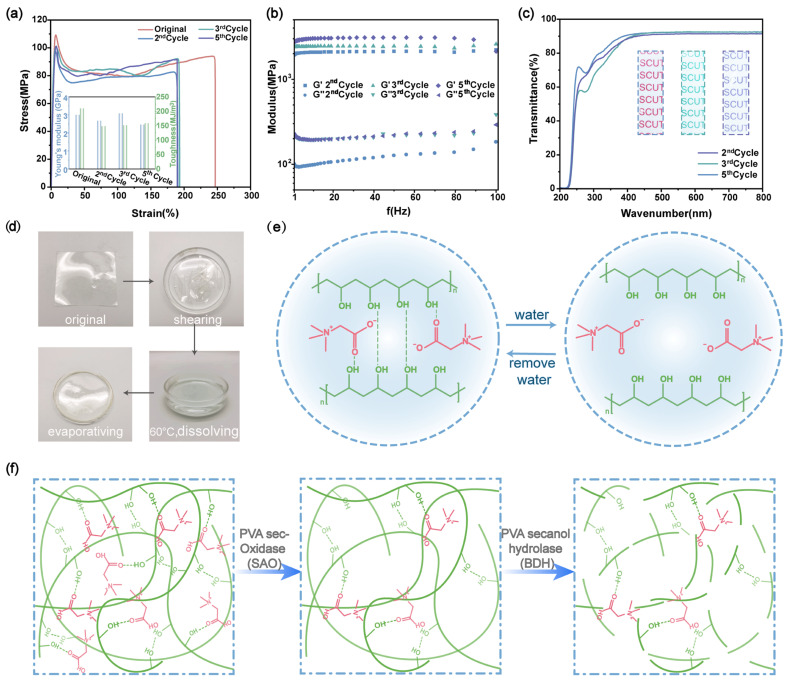
(**a**) Tensile stress–strain curves of the PVA/Bta supramolecular dry gel plastics after a different number of recycling cycles. The inset compares the mechanical properties of the original and recycled supramolecular dry gel plastics, (**b**) storage shear modulus (*G*′) and loss shear modulus (*G*″) of PVA/Bta as a function of frequency, and (**c**) transmission spectra and optical photographs of the initial and recycled PVA/Bta films. (**d**) Graphical representation and (**e**) schematic of the dissolution and regeneration of the PVA/Bta10% films, and (**f**) schematic of the degradation of the PVA/Bta dry gel plastics in soil.

The crosslinking between PVA and Bta is based on hydroxyl and carbonyl ester bonds and is thus reversible. This and the good solubility of PVA and Bta in water enable the closed-loop regeneration of the prepared plastics in an aqueous environment. The closed-loop recycling of PVA/Bta plastics is presented in Figure 4d,e. The PVA/Bta plastics are broken and put into a Petri dish filled with deionized water, resulting in plastic dissolution. Water molecules then penetrate between the PVA molecules, increasing their molecular spacing and breaking the structural hydrogen bonds in certain crystalline regions, exposing more free –OH. This causes the PVA chain segments to reversibly disentangle, forming polymer segments that are diluted, dispersed, or dissolved in water. When the water evaporates, the linkages and entanglement of the PVA chain segments are restored [1]. Following five successive fatigue cycles, its tensile strength and transparency in each cycle can reach 92% and 100% of the original PALB supramolecular polymers, respectively (Figure 4a–c). Thus, the produced supramolecular polymers exhibit improved sustainability.

When assessing the environmental friendliness of polymeric materials, biodegradability is a crucial metric. After traditional cultivation, PVA/Bta shows high biodegradability in natural soil. The pathway of PVA/Bta degradation in natural soil is presented in Figure 4f. In the initial stage, the mixed microbial community present in the natural soil gradually attaches to the film surface, and PVA/Bta is decomposed into fragments at the macroscopic level. Subsequently, the film fragments are broken into polymer chains, which are further decomposed into small molecules that are consumed by the soil microbial community [11,49,58]. At the same time, PVA has a high water-retaining capacity, and its hydrophilic functional groups increase the involved soil hydrophilicity; thus, the breakdown of PVA may benefit the soil [59].

### 2.4. Solvent Resistance of the PVA/Bta Dry Gel Plastics

**Figure 5:**
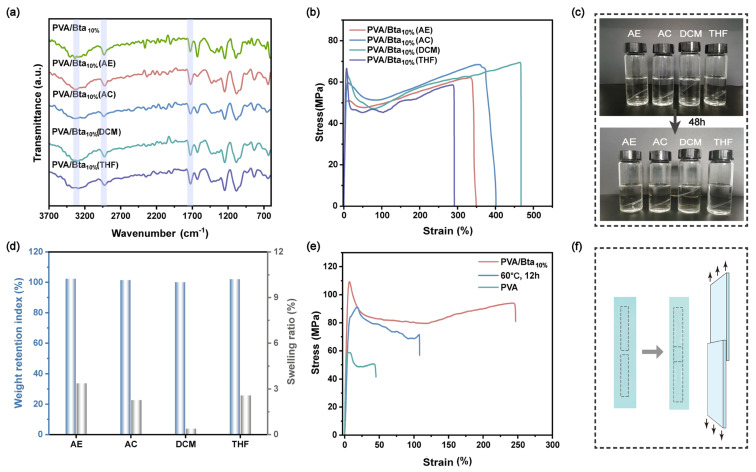
PVA/Bta10% in ethanol, acetone, dichloromethane, and tetrahydrofuran after 48 h. (**a**) FTIR spectra, (**b**) stress–strain curves, (**c**) comparison of physical photos, and (**d**) dissolution and weight retention indices. Thermal recycling of the PVA/Bta supramolecular dry gel plastics: (**e**) stress–strain curves, and (**f**) diagram of the physical operation.

PVA/Bta supramolecular dry gel plastics can be recycled multiple times and maintain their stability in most organic solvents. After a 48 h incubation in four solvents (ethanol, acetone, dichloromethane, and tetrahydrofuran) at room temperature, the transmission peak positions of the supramolecular polymers and the number of these peaks do not change (Figure 5a). The FTIR characterized the plastics after soaking in different solvents. The absorption peaks at 1628 cm^−1^, 2931 cm^−1^, and 3299 cm^−1^ indicate that the carbonyl (C=O) bond in the original Bta structure and the methylene(-CH_2_) and hydroxyl (-OH) bonds in the PVA structure have not been disrupted. Compared to the FTIR spectrum of the plastics without soaking in different solvents (Figure 3a), the figure shows no new characteristic peaks forming, confirming that the supramolecular system still only has noncovalent physical crosslinking after soaking in different solvents. After soaking in four solvents, AE, AC, DCM, and THF, the tensile strength of the plastic was 51.03 MPa, 59.90 MPa, 64.93 MPa, and 66.46 MPa, respectively. Compared with the plastic without solvent soaking, the tensile strength decreased by 53%, 45%, 40%, and 39%. The tensile strains of the plastic soaked in solvent were 335%, 371%, 465%, and 288%, respectively, which increased by 36%, 51%, 89%, and 17% compared to the untreated plastic. This implies that the supramolecular dry gel plastics may retain their structure and their initial mechanical strength after exposure to organic solvents (Figure 5b). Figure 5c shows the actual photos of the samples soaked in various solvents for 48 h. Furthermore, Figure 5d shows that the weight index is maintained at ~100%, suggesting that the plastic films do not lose weight during solvent immersion. Additionally, the swelling rate remains around 4% (relative to the weight of the solvent sample without solvent immersion), which is essentially negligible, and its shape and structural integrity are preserved.

The PVA/Bta supramolecular dry gel plastics also exhibit water-induced plasticization because of the abundant hydrogen-bonding crosslinking, enabling the rapid recycling of the dry gel plastics after breaking. Water may easily link hydrogen-bonding connections in the supramolecular plastic system because of the dynamic nature of hydrogen bonds. Using a razor blade, pieces of the PVA/Bta film (60 mm × 10 mm × 0.2 mm) were sliced into two halves. The ends of the two halves were dipped in water for 10 s, overlapped, and heated at 60 °C under pressure for 2 h, resulting in the reconnection of the two halves. In this manner, various components from the PVA/Bta plastics may be connected and recombined. The reconnected PVA/Bta plastics exhibit tensile strength similar to that of the original plastics (Figure 5e), with the stress remaining at 90 MPa, the strain reaching 105%, and the toughness remaining at 81 MJ m^−3^, substantially higher than those of pure PVA plastics and in the research of Wu et al. (only 60 MJ m^−3^) [54]. In the presence of water and heat, the hydrogen bonds in the PVA/Bta plastics can undergo dynamic fracture. At the plastic contact surface, PVA chains diffuse into one another and reform their hydrogen bonds with Bta, forming a secure linkage after cooling to room temperature. Figure 5f illustrates the physical operations of cutting the sample and reconnecting it.

## 3. Conclusions

Degradable PVA/Bta dry gel plastics with a high mechanical strength and good tensile properties were obtained by introducing water to prepare PALBs using PVA as an HBD and natural nontoxic quaternary ammonium salt Bta as an HBA. The system comprises dense intermolecular forces, such as hydrogen bonding, which are achieved by adjusting the mass ratio of Bta to achieve the regulation of the overall properties of PVA/Bta dry gel plastics. When Bta is brought up to 10%, PVA/Bta10% possesses both strong mechanical strength (109 MPa) and specific tensile characteristics (~256%). Furthermore, these PVA/Bta supramolecular polymers exhibit high transparency (~92%), are recyclable, and degrade autonomously in soil. Due to the simplicity of the preparation technique of PVA/Bta10% dry gel plastics, no complex equipment is required. Thus, we suggest that PVA/Bta10% dry gel plastics can lessen the harm to the environment that comes from excessively using nonbiodegradable dry gel plastics.

## 4. Materials and Methods

### 4.1. Materials

PVA (1799 type, 98–99% alcoholysis, and 23–31 mPa·S viscosity) and Bta (98%) were purchased from McLean Biochemical Co., Shanghai, China.

### 4.2. Polymer Preparation

The PVA (2 g) and Bta (0.1 g) were dissolved in 25 mL of deionized water. The resultant solution was heated in an oven at 85 °C for 36 h to remove most of the involved water to obtain a crude product. This crude product was dried in a vacuum oven at room temperature until a constant mass was achieved, yielding a deep eutectic supramolecular polymer. Considering the number of hydroxyl and carboxyl groups, the involved PVA:Bta mass ratios were 20:1, 10:1, 6.7:1, and 5:1. The mass fractions of Bta were obtained as (Bta mass/PVA mass) × 100%. The remaining supramolecular polymers were prepared according to the above method.

### 4.3. Characterization

Differential scanning calorimetry (DSC; TOLEDO DSC3, METTLER, Switzerland, Zurich) was conducted in a nitrogen atmosphere at a heating rate of 10 °C min^−1^ in a temperature range of −50–200 °C. The Fourier transform infrared (FTIR) spectra were measured using an attenuated total reflectance FTIR spectrometer (Nicolet IS50—Nicolet Continuum, Thermo Fisher Scientific, Waltham, MA, USA) in a wavelength range of 500–4000 cm^−1^. The ^1^H NMR spectra of the PALBs were obtained using the AVANCE III HD 600 (Bruker, Karlsruhe, Germany).

The transmittance was measured in a range of 200–800 nm using a UV–visible spectrophotometer (UV-2600i, Kyoto, Japan). The X-ray diffraction patterns were measured using an X’pert Powder instrument (PANalytical, Almelo, The Netherlands).

The surface morphology of the PALBs was evaluated after plating, and the surface morphology of the involved plastics was observed using a Zeiss Merlin instrument at 25 °C and 30% humidity. The morphology of the PALBs was further analyzed using atomic force microscopy (Dimension Icon, Bruker, Karlsruhe, Germany). The thermal stability of the PALBs was determined using a thermogravimetric analyzer (TG209F3, NETZSCH, Selb, Germany) in a nitrogen atmosphere from 50 °C to 600 °C at a heating rate of 10 °C min^−1^.

The mechanical properties of the dry gel plastics were tested at a constant speed of 10 mm min^−1^ using a universal testing machine (Instron 5565, INSTRON, Boston, MA, USA) with a 500 N compression element. Uniaxial tensile tests were performed on rectangular samples (30 mm × 10 mm × 0.2 mm). The storage modulus (*G*′), loss modulus (*G*″), and loss factor (tanδ) of the prepared dry gel plastics were evaluated via dynamic thermomechanical analysis (DMA, TA, New Castle, DE, USA) to analyze the plastics’ viscoelastic behavior. This analysis was conducted on samples with a length of <30 mm, a width of 10 mm, and a thickness of 0.2 mm. The relation between the PVA/Bta modulus and the temperature was determined in a temperature range of −20–120 °C at a frequency of 1 Hz and a heating rate of 3 °C min^−1^ in the variable-temperature single-frequency stretching mode. The relation between the PVA/Bta modulus and the frequency was determined in the constant-temperature variable-frequency stretching mode and a frequency range of 0–100 Hz at 25 °C. The calculation method for the weight retention index was ${\;W}_{2}=\frac{W_{1}}{W_{0}}*100\%$, where W_2_ represents the weight retention index (%), W_1_ represents the weight of the sample immersed in solvent and then dried (g), and W_0_ represents the weight of the sample without solvent immersion (g). The swelling ratio was $S_{2}=\frac{S_{1}}{S_{0}}*100\%$, where S_2_ represents the swelling ratio (%), S_1_ represents the weight of the sample immersed in solvent and then driedthe weight of the sample without solvent immersion (g), and S_0_ represents the weight of the sample without solvent immersion (g).

All the calculations were performed using the Gaussian 09 software package. Geometric optimization was conducted using the B3LYP functional with the 6–31G(D) basis group. The DFT-D3(BJ) dispersion correction was employed to correct for the medium- and long-range van der Waals interactions [60]. In addition, the singlet point energy of each structure at the M06–2X/Def2TZVP level was calculated [61]. The basis set superposition error correction was performed in the calculations of the interaction energies of the optimized complexes. To gain insight into the intermolecular interactions, independent gradient modeling was performed on the structure of the involved complexes using Multiwfn software 3.7 [62,63,64]. The corresponding color-filled isosurface plots were generated using the Visual Molecular Dynamics software 1.9.4 [65]. The plan was carried out in a gas-phase environment.

## Data Availability

The data supporting this article have been included as part of the ESI.

## Supplementary Materials

The following supporting information can be downloaded at https://www.mdpi.com/article/10.3390/gels11060421/s1, Figure S1: preparation of supramolecular dry gel plastic PVA/Bta.; Figure S2: comparison of Young’s modulus and toughness of PVA/Bta supramolecular dry gel plastics; Figure S3: (a) comparison of melting points of PVA and PVA/Bta10%, (b) FTIR spectra of PVA/Bta, and (c) 1H NMR comparison before and after PVA/Bta10% preparation; Figure S4: ball-and-stick model of PVA/Bta, (a) molecular independent gradient model isosurfaces of PVA and PVA/ChClDEP blends, (b) atomic colors: H: white; O: red; N: blue; and C: gray; Table S1: fabrication of PVA/Bta blends; Table S2: strength of the binding potential between the different components; Table S3: Heat loss parameters for PALB plastics.

## Abbreviations

The following abbreviations are used in this manuscript:

DSC	Differential scanning calorimetry
HBA	Hydrogen bond acceptor
HBD	Hydrogen bond donor
PDES	Polymerizable deep eutectic solvent
PALBs	PVA/Bta dry gel plastics
AA	Acrylic acid
ChCl	Choline chloride
