# Supplementary material for: Recyclable and Degradable Poly(vinyl alcohol)/Betaine-Based Deep Eutectic Polymer Dry Gel Plastics with a High Mechanical Strength
Source: Gels. 2025 May 31;11(6):421. doi: 10.3390/gels11060421 (PMC12192343; doi:10.3390/gels11060421)
Supplement: Supplementary file 1 [file gels-11-00421-s001.zip › gels-3609327-supplementary.pdf]

Supporting Materials

Recyclable and Degradable Poly(Vinyl Alcohol)/Betaine-Based Deep Eutectic Polymer Dry Gel Plastics with a High Mechanical Strength

Hanyu Zhao [1,†], Ying Jia [1,†], Ling Cai [2], Xiaochun Wang [1] and Minghui He [1] and Guangxue Chen [1,3,*]

[1] State Key Laboratory of Pulp and Paper Engineering, School of Light Industry and Engineering, South China University of Technology, Guangzhou 510640, China
[2] Division of Engineering in Medicine, Department of Medicine, Brigham and Women's Hospital, Harvard Medical School, Cambridge, MA, 02139 USA
[3] Guangdong Province Filtration and Wet Nonwoven Composite Materials Engineering Technology Research Center, Guangzhou 510640, China
† Zhao Hanyu and Jia Ying contributed equally to this work.
* Correspondence: Corresponding author's E-mail: chengx@scut.edu.cn

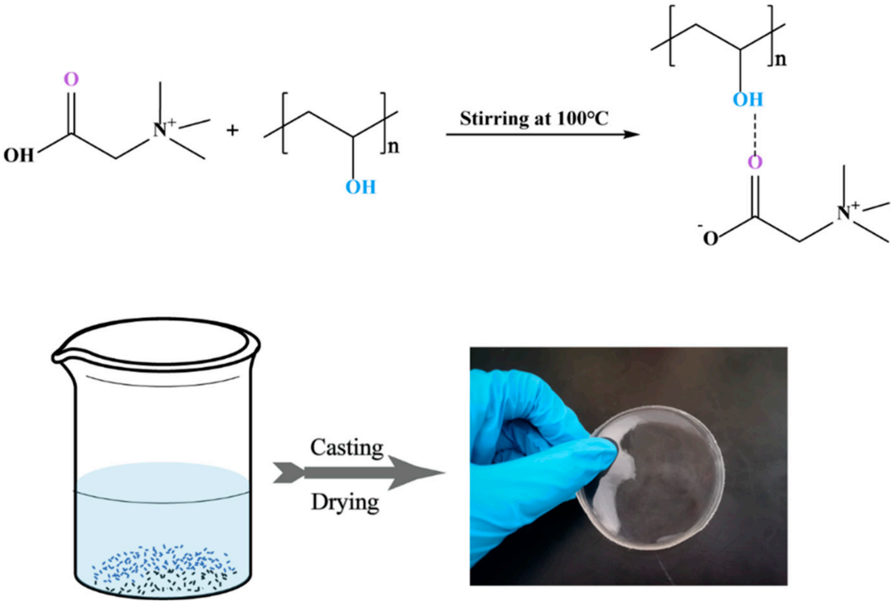

Figure S1. Preparation of supramolecular dry gel plastic PVA/Bta.

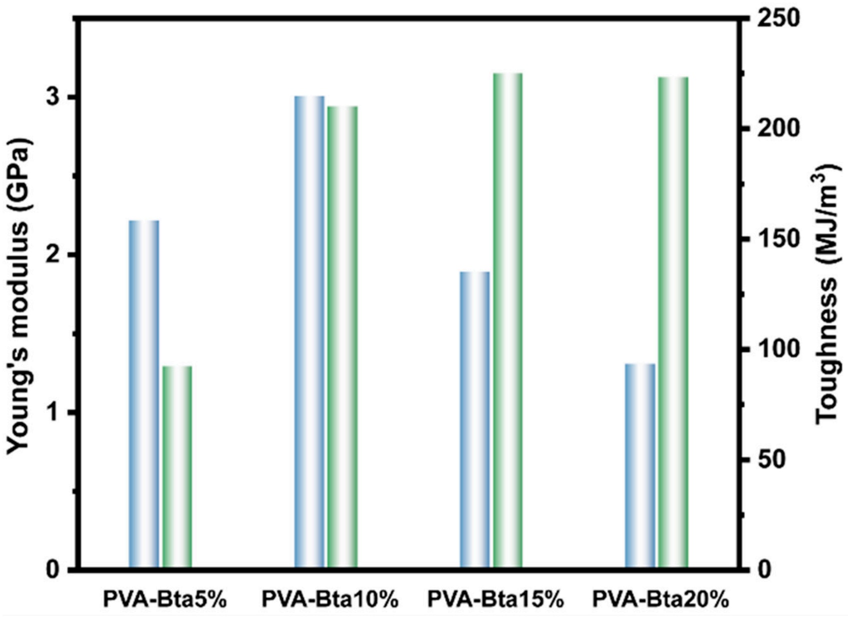

Figure S2. Comparison of Young's modulus and toughness of PVA/Bta supramoleculsar dry gel plastics.

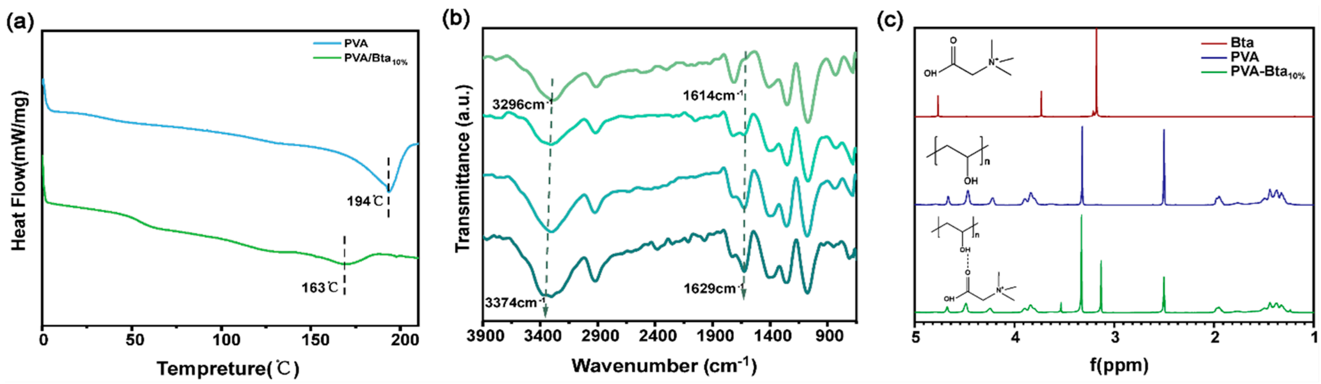

Figure S3. (a) Comparison of melting points of PVA and PVA/Bta10%, (b) FTIR spectra of PVA/Bta, and (c) 1HNMR comparison before and after PVA/Bta 10% preparation.

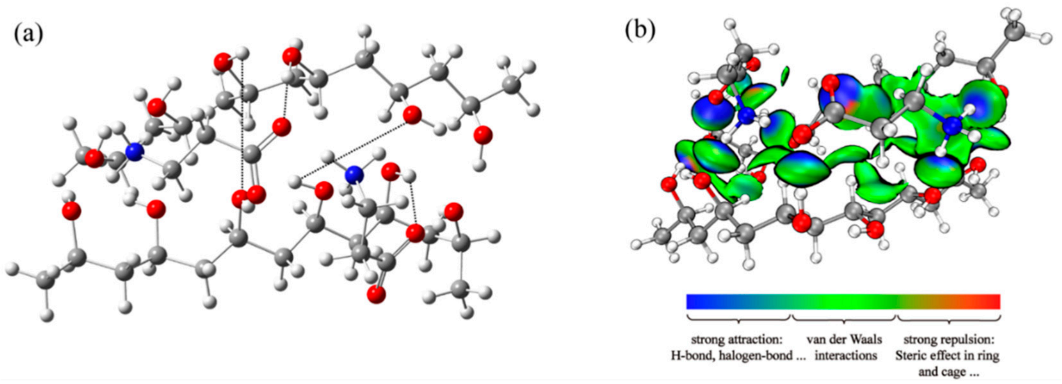

Figure S4. Ball-and-stick model of PVA-Bta.(a), Molecular independent gradient model (IGM) isosurfaces of PVA and PVA/ChClDEP blends(b). Atomic colours: H: white; O: red; N: blue; C: grey.

Table S1. Fabrication of PVA/Bta Blends.

Code	PVA (g)	Betaine(g)	Deionized Water (mL)
PVA/$Bta_{5\%}$	2	0.1	25
PVA/$Bta_{10\%}$	2	0.2	25
PVA/$Bta_{15\%}$	2	0.3	25
PVA/$Bta_{20\%}$	2	0.4	25

Table S2. Strength of the binding potential between the different components.

Compound1	Compound2	E(Kcal/mol)
PVA	PVA	-21.53
PVA	Bta	-31.59

Table S3. Heat loss parameters for PALB plastics.

PALB	T_{onset} (℃)	T_{max} (℃)	Maximum degradation rate (wt%/℃)
$PALB_{5\%}$	218.1	330.1	6.2
$PALB_{10\%}$	216.6	325.4	5.6
$PALB_{15\%}$	215.1	318.9	5.4
$PALB_{20\%}$	213.3	314.7	5.3
